# Improvement of Resistance Change Memory Characteristics in Ferroelectric and Antiferroelectric (like) Parallel Structures

**DOI:** 10.3390/nano13030439

**Published:** 2023-01-21

**Authors:** Wonwoo Kho, Hyunjoo Hwang, Jisoo Kim, Gyuil Park, Seung-Eon Ahn

**Affiliations:** 1 Department of IT ∙ Semiconductor Convergence Eng, Tech University of Korea, Siheung 05073, Republic of Korea; 2 Department of Nano & Semiconductor Eng, Tech University of Korea, Siheung 05073, Republic of Korea

**Keywords:** ferroelectric, antiferroelectric (like), multiresistance state

## Abstract

Recently, considerable attention has been paid to the development of advanced technologies such as artificial intelligence (AI) and big data, and high-density, high-speed storage devices are being extensively studied to realize the technology. Ferroelectrics are promising non-volatile memory materials because of their ability to maintain polarization, even when an external electric field is removed. Recently, it has been reported that HfO_2_ thin films compatible with complementary metal–oxide–semiconductor (CMOS) processes exhibit ferroelectricity even at a thickness of less than 10 nm. Among the ferroelectric-based memories, ferroelectric tunnel junctions are attracting attention as ideal devices for improving integration and miniaturization due to the advantages of a simple metal–ferroelectric–metal two-terminal structure and low ultra-low power driving through tunneling. The FTJs are driven by adjusting the tunneling electrical resistance through partial polarization switching. Theoretically and experimentally, a large memory window in a broad coercive field and/or read voltage is required to induce sophisticated partial-polarization switching. Notably, antiferroelectrics (like) have different switching properties than ferroelectrics, which are generally applied to ferroelectric tunnel junctions. The memory features of ferroelectric tunnel junctions are expected to be improved through a broad coercive field when the switching characteristics of the ferroelectric and antiferroelectric (like) are utilized concurrently. In this study, the implementation of multiresistance states was improved by driving the ferroelectric and antiferroelectric (like) devices in parallel. Additionally, by modulating the area ratio of ferroelectric and antiferroelectric (like), the memory window size was increased, and controllability was enhanced by increasing the switchable voltage region. In conclusion, we suggest that ferroelectric and antiferroelectric (like) parallel structures may overcome the limitations of the multiresistance state implementation of existing ferroelectrics.

## 1. Introduction

According to the real-time statistics site ‘Worldometers’, presently, there are more than 5.4 billion Internet users around the world, and more than 280 billion emails are exchanged per day. In addition, IDC, a U.S. market intelligence firm, has also predicted that by 2022, the total volume of digital information will reach 80 ZB (1 trillion GB is 1 ZB) [1]. As the world is currently accumulating and utilizing a vast amount of data, there is a need for breakthrough improvements in storage devices. Ferroelectric materials are promising for memory applications due to their non-volatile properties [2,3]. However, conventional perovskite ferroelectrics have the limitation of losing ferroelectricity as their thickness decreases [4,5].

Doped HfO_2_-based ferroelectric materials have received tremendous attention since the discovery of ferroelectricity in the TiN–HfO_2_–TiN structure of the 10 nm 3% Si-doped HfO_2_ film in 2011 [6]. Doped HfO_2_-based ferroelectric can maintain ferroelectricity even at low thicknesses because the doped HfO_2_-based ferroelectric has a high dielectric constant (~25) and large bandgap (~5.8 eV) [7], and has the significant benefit of being easily compatible with existing complementary metal–oxide–semiconductor (CMOS) processes. HfO_2_-based thin films exist in a mixed state of several phase states [8]. The following phases may exist [9]: cubic phase (Fm$\bar{3}$m, c-phase), tetragonal phase (P4_2_/nmc, t-phase), monoclinic phase (P2_1_/c, m-phase), orthorhombic I phase (pbca, o-phase), orthorhombic II phase (pnma, oII-phase), and polar orthorhombic III phase (Pca2_1_, f-phase). There has been an increase in interest in the relationship between various phases in the m-phase, t-phase, and especially the f-phase, following many reports suggesting that the ferroelectricity of HfO_2_ is based on the f-phase, which is a non-centrosymmetric polar crystal phase [10]. According to several experimental findings, the f-phase, known as the second stable phase, is observed between the m-phase and t-phase [11,12]. The phases in HfO_2_-based thin films may differ depending on various factors such as the temperature, dopant, doping concentration, stress, strain, surface energy, interface, and size effect. This is because there is a minor difference in the free energies of these phases [13]. In particular, the energy difference is significantly small at just 1 meV/f.u. between the f- and t-phases of the HfO_2_-based thin films. In addition, the HfO_2_-based thin films exist in a mixed state of several phases, so they may be antiferroelectric (like), in which ferroelectricity by the f-phase and antiferroelectricity by the t-phase are simultaneously expressed. Recent findings show that an HfO_2_-based thin film with a thickness of less than 8 nm transitions from ferroelectric to antiferroelectric, which can be explained by the surface energy effect [14]. The antiferroelectric (like) has a non-zero remnant polarization (P_r_) and double hysteresis loop characteristic due to the mixing of the double hysteresis loop characteristic of antiferroelectrics and maintenance of the polarization characteristics of ferroelectrics [15]. The polarization–voltage (P–V) hysteresis loop of antiferroelectrics (like) is also called the broken hysteresis loop, and most importantly, antiferroelectrics (like) have a non-volatile property because P_r_ is not zero.

The ferroelectric tunnel junction (FTJ) has been evaluated as a promising memory device, driven by P_r_ [16]. It has an advantage in power consumption due to the tunneling mechanism, and its simple metal–ferroelectric–metal (MFM) structure has excellent integration. The tunneling current of the FTJ can be adjusted by modulating the potential barrier according to the polarization direction and the amount of P_r_. Depending on the direction of polarization, the ‘on’ state, in which a large amount of current flows is called the low resistance state (LRS), and the ‘off’ state, in which a small amount of current flows, is called the high resistance state (HRS) [17]. The manifestation of these two states is called the tunneling electroresistance (TER) effect. An asymmetric potential barrier must arise due to different screening lengths of the top and bottom electrode interfaces to express the TER effect in the FTJ. An asymmetric potential barrier may appear, even when the same metal is used for the top and bottom electrodes. This is explained by the TiO_x_N_y_ layer formed on the interface between the electrode and ferroelectric layer using TiN equally for the top and bottom electrodes, as shown in previous studies [13,18]. By partially switching the polarization between the HRS and LRS, the FTJ can implement multiresistance states, and the partial polarization switching can be adjusted in accordance with the amplitude and width of the applied pulse [19,20]. Under the same principle, even if an antiferroelectric (like) layer is applied to the FTJ instead of a ferroelectric layer, a TER effect may occur because P_r_ is not 0 and may still be driven in the same way as a general FTJ [21]. Although the operating method was the same in both cases, there were differences in the polarization switching characteristics as ferroelectric and antiferroelectric (like) have one and two coercive fields in one polarity, respectively [22]. It is anticipated that the memory performance will be increased through a broad and/or wide switching field, if the ferroelectric and antiferroelectric (like) with these other polarization switching characteristics are driven in parallel.

To present a next-generation memory device that can store more information in a single memory cell, in this study, a ferroelectric and antiferroelectric (like) parallel structure-based FTJ device was proposed. The multiresistance states measured in this parallel structure were analyzed to confirm the increased memory window, and controllability was confirmed compared to the existing FTJ. In addition, the best area ratio was extracted by analyzing the implementation of the multiresistance states according to the area ratio of the ferroelectric and antiferroelectric (like). This result suggests that the performance of the resistance change memory can be enhanced by the parallel structure of the ferroelectric and antiferroelectric (like).

## 2. Materials and Methods

**Figure 1:**
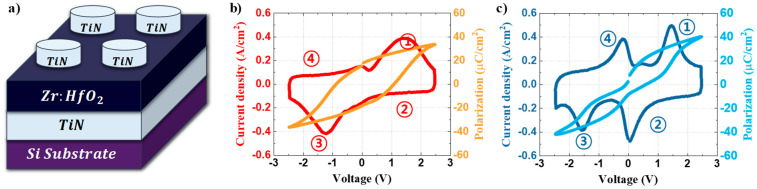
Schematic representation and electrical properties of the TiN/Hf_0.35_Zr_0.65_O_2_/TiN devices: (**a**) TiN/Hf_0.35_Zr_0.65_O_2_/TiN device structure; (**b**) electrical properties of the TiN/10 nm-thick Hf_0.35_Zr_0.65_O_2_/TiN device (red line denotes the pulsed J–V curve and orange line denotes the P–V curve); (**c**) electrical properties of the TiN/6 nm-thick Hf_0.35_Zr_0.65_O_2_/TiN device (blue line denotes the pulsed J–V curve and sky-blue line denotes the P–V curve).

Ferroelectric and antiferroelectric (like) thin films were grown via the ALD (atomic layer deposition) process. On a TiN/SiO_2_/Si substrate, Zr doped HfO_2_-based ferroelectric and antiferroelectric (like) layers were deposited via the thermal-ALD process at 300 °C. According to the Hf:Zr ratio and film thickness in the Hf_x_Zr_1−x_O_2_(HZO) system, the ferroelectric or antiferroelectric (like) can be stabilized [23,24]. Cocktail precursor having a molar ratio 0.35:0.65 of cyclopentadienyl-tris(dimethylamino)-hafnium (Hf[Cp(NMe_2_)_3_]) and cyclopentadienyl-tris(dimethylamino)-zirconium (Zr[Cp(NMe_2_)_3_]) was used, and ozone was used as the reactant gas. Hf_0.35_Zr_0.65_O_2_ thin films were deposited in thicknesses of 10 nm and 6 nm, respectively, to obtain ferroelectric and antiferroelectric (like) properties. Then, a top TiN electrode with a thickness of 60 nm was deposited using a reactive sputter and a shadow mask in a circular pattern with a diameter of 200 µm. Finally, a HZO device with an MFM structure, as shown in Figure 1a, was created by performing rapid thermal annealing at 600 °C for 60 s in a N_2_ environment.

## 3. Results

The ferroelectricity and antiferroelectricity (like) of the fabricated capacitor were demonstrated using a semiconductor parameter analyzer (Keithley 4200 with a 4225-PMU module). The current density-voltage (J–V) curve was extracted by considering the area of the top electrode in the current acquired by applying the triangular pulse at a frequency of 1 kHz. The polarization–voltage (P–V) curve was then derived from the pulsed J–V curve. The red line in Figure 1b depicts the J–V curve of a TiN/10 nm-thick Hf_0.35_Zr_0.65_O_2_/TiN capacitor, and the order of polarization switching is as follows. ① Switching occurs gradually according to the applied voltage; ② switched polarization is maintained even if the applied voltage is gradually decreased and removed, which can be observed from the fact that there is no transient current peak, in which the polarization is switched in the opposite direction; ③ maintained polarization is switched in the opposite direction again when a voltage of the opposite polarity is applied; and finally ④, the polarization direction is maintained, as in ②, even if the applied voltage is gradually removed, which is a typical ferroelectric characteristic. Additionally, the TiN/10 nm-thickness-Hf_0.35_Zr_0.65_O_2_/TiN capacitors had a general ferroelectric hysteresis loop, as shown by the P–V curve, depicted as an orange line in Figure 1b. The blue line in Figure 1c depicts the J–V curve of a TiN/6 nm-thick Hf_0.35_Zr_0.65_O_2_/TiN capacitor, and the order of polarization switching is as follows. ① Switching occurs gradually according to the applied voltage; ② a certain amount of switched polarization is switched back in the opposite direction as the applied voltage is gradually removed, however, ferroelectricity remains because P_r_ is not zero at 0 V, from which the external electric field has been removed; ③ when a voltage of the opposite polarity is applied, the remaining polarization is easily switched, even at a low voltage near 0 V, and switching occurs gradually as the voltage is applied further.; and ④ when the applied voltage is gradually removed, a certain amount of switched polarization is switched again in the opposite direction, as shown in ②. The sky-blue line in Figure 1c is the P–V curve derived from the J–V curve. The fabricated TiN/6 nm-thick-Hf_0.35_Zr_0.65_O_2_/TiN capacitor is an antiferroelectric (like) with a double hysteresis loop, which is an antiferroelectric characteristic, and a non-zero P_r_, which is a ferroelectric characteristic. It was confirmed that the fabricated TiN/10 nm-thick-Hf_0.35_Zr_0.65_O_2_/TiN and TiN/6 nm-thick-Hf_0.35_Zr_0.65_O_2_/TiN capacitors were ferroelectric and antiferroelectric (like), respectively.

The FTJ is driven by adjusting the tunneling electrical resistance by modulating the potential barrier in accordance with P_r_ [25,26]. P_r_ can be adjusted by using partial polarization switching. Multiresistance states were obtained in the range between the HRS and LRS through the TER effect. Even if an antiferroelectric (like) is applied instead of a ferroelectric, it may be used in a manner similar to the FTJ as P_r_ is not zero and there is the possibility of gradual polarization switching. C-AFM measurements of AFM were conducted to demonstrate the TER effect [27], which is the basis of the Fowler–Nordheim tunneling (FNT) mechanism [28]. The C-AFM mode extracts the current distribution by contacting and scanning a conductive cantilever with a sample. The measurements were performed in an HZO/TiN structure, and a conductive cantilever was used as the upper electrode. The current distribution was extracted after the domains were aligned in reverse by applying voltages with different polarities to each region. Each region’s domain alignment was determined by the movement of the conductive cantilever used as the top electrode, while voltage was applied to the bottom electrode. The measurement procedure was performed under the same conditions for both manufactured devices.

**Figure 2:**
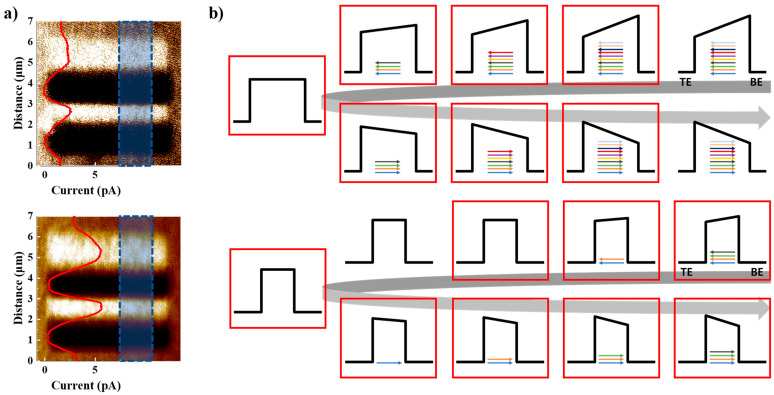
Verification of the TER effects and description of memory characteristics through a schematic of the energy band diagram. (**a**) C-AFM image of the HZO/TiN structure: ferroelectric (upper panel) and antiferroelectric (like) (lower panel); “the insertion data” (red line) is a mapped current extracted from the blue square area; (**b**) schematic of the p_r_ control in accordance with the applied pulse of ferroelectric (upper panel) and antiferroelectric (like) (lower panel).

The current for the two aligned regions and the pristine region was then extracted in a region 7 m × 7 m larger than the region, to which the voltage was already applied. A high-performance ultralow current amplifier (ULCA) (Park systems) was used for the exact current extraction. A mapped current–distance image of the ferroelectric material is shown in the top panel of Figure 2a. It is evident from the color that the current levels in the area where −8 V was applied and area where +10 V was applied were different. The red line in the inset of Figure 2a is the current extracted from the blue square area. The current levels were approximately 0.0 pA and 2.4 pA, respectively, in the regions where −8 V and +10 V were applied in advance. These current levels were different to the 1.5 pA extracted from the pristine region. The antiferroelectric (like) corresponding to the lower panel of Figure 2a exhibited the same tendency as the ferroelectric. The current levels were approximately 0.3 pA and 5.4 pA, respectively, in the regions where −8 V and +10 V were applied in advance, and these current levels were different from the 2.9 pA extracted from the pristine region. In both cases, the TER effect was visible with a different current level depending on the voltage applied in advance at the same scan voltage. This implies that the manufactured ferroelectric and antiferroelectric (like)-based devices can be operated as FTJs. In addition, as the measurement results show, ferroelectric and antiferroelectric (like) materials have different levels of current distribution. Wider or numerous transient current peaks can be achieved by combining the switching properties of the two devices. This may provide an advantage in implementing multiple states of resistance-change memory. Figure 2b shows a schematic of P_r_ control in accordance with an external signal, illuminating the differences between the switching properties of a ferroelectric and an antiferroelectric (like) in terms of potential barrier modulation. In this figure, TE and BE mean the top and bottom electrodes; the position is reported horizontally; the energy vertically, and the arrows represent the p_r_. In both cases, the process of pointing to the lower electrode was demonstrated by starting with the polarization pointing to the upper electrode and gradually switching the polarization in the opposite direction. The schematic, which is represented as an energy band diagram, for this process contains the following assumptions and conditions. First, the polarizations of both ferroelectrics and antiferroelectrics (like) were polarized in a direction perpendicular to the electrode. Second, the P_r_ of the ferroelectric was expressed with ten arrows, and the antiferroelectric (like) was expressed with four arrows, which was 40% of the ferroelectric. This was the approximate amount assumed from the P_r_ value of the P–V curve shown in Figure 1b,c. Third, only the remaining P_r_ was considered after offsetting the polarization in the opposite direction. A schematic of the ferroelectric polarization switching process when a larger voltage is applied over time is shown in the upper panel of Figure 2b. Similar to the transient current response of the first quadrant of Figure 1b, when the applied external signal was small (when applied about 0.2 V or less), there was no change in the aligned polarization in the initial stage. The aligned polarization was then switched to the opposite direction when additional external signals were applied (when applied more than about 0.2 V), offsetting and reducing the total P_r_. Subsequently, as more external signals were applied, the polarization aligned in a direction opposite to the initial state. A schematic of the antiferroelectric (like) polarization-switching process is shown in the lower panel of Figure 2b. Even when the applied external signal was small, the aligned polarization began to switch because the transient current peak spanned to zero, as shown in Figure 1c. In contrast to the ferroelectrics, polarization switching at this time did not switch in the opposite direction but in a non-polar phase. However, the nonpolar phase was not considered separately because it had no impact on the potential barrier. After polarization switching according to the external signal near 0 V, polarization switching occurred again at a specific voltage or higher. A red square was inserted in both the ferroelectric and antiferroelectric (like) states when the electrical resistance state was changed by polarization switching in accordance with the applied external signal. As described earlier, ferroelectric and antiferroelectric (like) materials exhibit different switching characteristics according to the external signals. Thus, the gradual switching characteristic may be improved when ferroelectrics and antiferroelectrics (like) are driven in parallel. This is expected to lead to an improvement in the performance of the resistance-change memory to implement multiresistance states.

**Figure 3:**
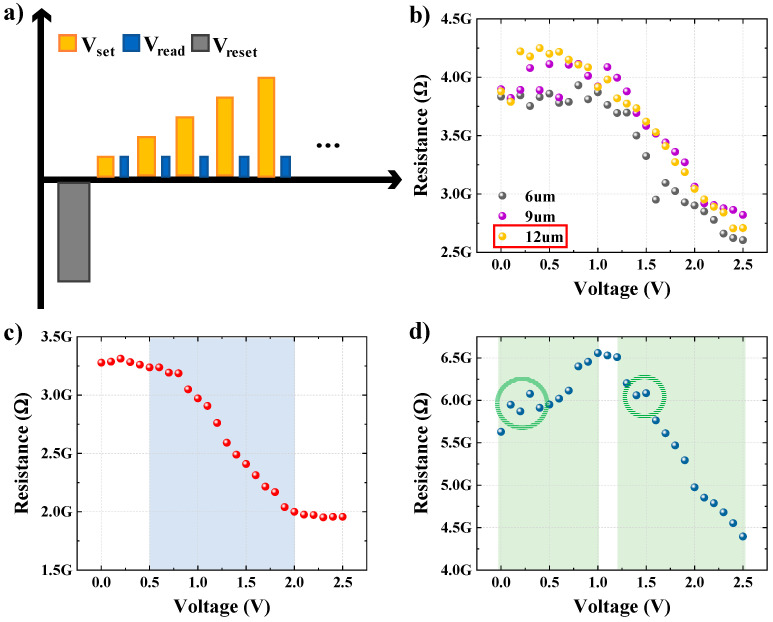
Schematic of the measuring sequence and implementation of multiple resistance states: (**a**) measuring sequences for the measurements of multiple resistance; (**b**) multiple resistance characteristics of parallel structures as a function of pulse width; (**c**) multiple resistance characteristics of the ferroelectric; (**d**) multiple resistance characteristics of the antiferroelectric (like).

The general techniques for implementing the various resistance states of the FTJ are amplitude or pulse width adjustments of the programming pulse [19,29]. Figure 3a depicts the measurement process, which gradually increases the amplitude of the programming pulse to change the resistance state. The resistance state, which depends on the programming pulse, can be calculated using the extracted current by applying the read voltage between the programming pulses. To measure the ferroelectric and antiferroelectric (like) in parallel, the measurement conditions must be set equally, and the conditions were set as follows. First, the same polling pulse must be set, so that the polarization of the ferroelectric and antiferroelectric (like) can be fully aligned equally. The amplitude of the polling pulse was set to −2.5 V and width to 100 μs after both cases were determined through the J–V curve shown in Figure 1b,c, which demonstrates that all switching was finished at 2.5 V. Second, the maximum amplitude (V_max_) of the programming pulse was set to 2.5 V for the same reason as the polling pulse, and it was applied by increasing from 0 V to V_max_ by 0.1 V steps. Third, the read voltage was set to −0.4 V to detect the tunneling current. Fourth, the conditions of 6 μs, 9 μs, and 12 μs were set to use a suitable width, and the measured characteristics of the multiresistance states can be confirmed from Figure 3b. It should be noted that the area of the top electrode doubles when the ferroelectric and antiferroelectric (like) are driven in parallel, as opposed to when only one is used. The resistance was plotted by considering that when the area doubled, the measured current level also doubled. The measured results show that when the width was 12 μs, a relatively large memory window was offered, and simultaneously, a relatively stable state was implemented. Here, the memory window is the value obtained by dividing the read voltage by the current measured from the read voltage of the device and refers to the difference of the LRS and the HRS that the resistance change memory may have at the same read voltage.

The individual ferroelectric and antiferroelectric (like) properties were also measured under the same conditions to further analyze the results measured in parallel. The multiresistance states of the ferroelectric and antiferroelectric (like) are shown in Figure 3c,d, respectively. Multiple resistance states were evaluated using two criteria, memory window size and controllability. First, the memory window should be evaluated, because the resistance state of the resistance change memory device can only have a value within the memory window. In addition, the multiple resistance state is controlled by polarization switching between the minimum voltage (V_min_) and maximum voltage (V_max_) at which polarization can be switched [30]. Accordingly, the difference between V_min_ and V_max_, which are the voltage ranges that affect polarization switching, was evaluated by considering the memory window size. The memory windows were 1.24 GΩ, 2.11 GΩ, and 1.47 GΩ for ferroelectricity, antiferroelectricity (like), and in parallel, respectively, which were the best when using antiferroelectricity (like) alone. Controllability was evaluated by simultaneously considering the memory window range and voltage area of the applied pulse in which the resistance changes. The ferroelectric began to show significant resistance level changes after 0.6 V and lasted until 2.0 V, and antiferroelectric (like) showed significant resistance level changes from 0 V to 1.0 V and 1.2 V to 2.5 V. This trend is consistent with that described in Figure 2b through the schematic of the energy band diagram (EBD). In the parallel measurement, a significant change in resistance level began after approximately 0.6 V and lasted until 2.5 V. Controllability was evaluated by extracting the change in resistance for a voltage change of 1 V, and the smaller this parameter is, the more advantageous it is for resistance control. The controllability parameters according to the memory window size were 0.88 ΔG/V, 1.63 ΔG/V, and 0.77 ΔG/V for the ferroelectric, antiferroelectric (like), and parallel structures, respectively, which were the best when using parallel structures. According to the aforementioned results, antiferroelectric (like) was the best in terms of memory window size, and the parallel structure was the best in terms of controllability. However, there was instability in the implementation of the resistance state when using the antiferroelectric (like) alone, as can be seen in the green circle in Figure 3d. Because of various issues such as stability and reliability, it is challenging to apply the antiferroelectric (like) alone to the resistance change memory. The parallel driving of the ferroelectric and antiferroelectric (like) has a larger memory window than the ferroelectric and can compensate for the stability issue of antiferroelectricity (like). In other words, a parallel structure is advantageous for implementing multiresistance states.

**Figure 4:**
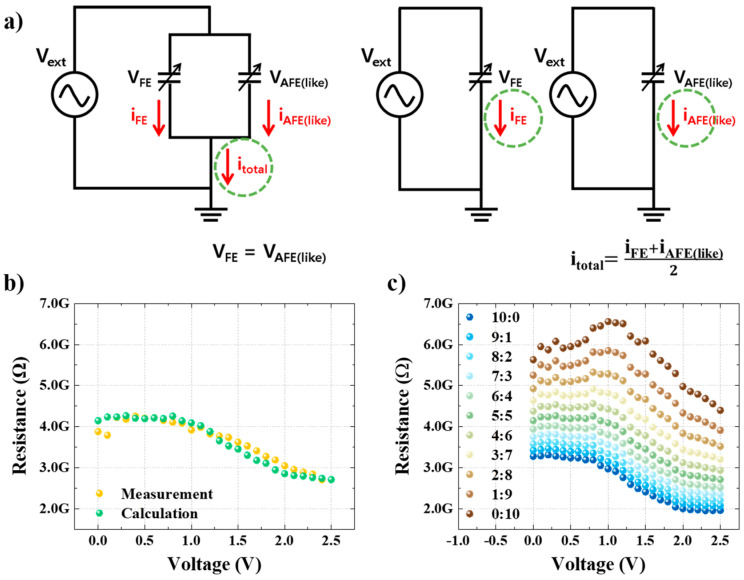
Schematic measurement circuit and resistance change curve according to the area ratio; (**a**) schematic of the measurement circuit (current measured in parallel (**left** panel) and separately (**right** panel)) (**b**) comparison of the measured parallel structural resistance state and individually measured and calculated resistance state; (**c**) resistance change curve calculated using the area ratio.

Because a ferroelectric and an antiferroelectric (like) were simultaneously measured in parallel, the same signal was applied, as shown in Figure 4a. The resistance state of each element programmed using the same signal was also extracted using the same read voltage. Accordingly, the results of measuring the ferroelectric and antiferroelectric (like) in parallel and applying the current derived from each measurement of ferroelectric and antiferroelectric (like) in an area ratio of 1:1 should ideally be equivalent. To confirm this, the resistance states obtained by the measurement and calculation were compared, and it was confirmed that they were nearly identical (Figure 4b). In other words, it is possible to extract a multiresistance state characteristic curve based on the area ratio of the results of individually measuring the ferroelectric and antiferroelectric (like) ratios. Figure 4c shows the resistance states of the ferroelectric and antiferroelectric (like) parallel structures with area ratios of 10:0, 9:1, 8:2, …, 0:10. It can be seen that the memory window size and controllability improved as the ratio of antiferroelectrics (like) increased; however, the stability problem also increased, as illustrated in Figure 3d.

**Figure 5:**
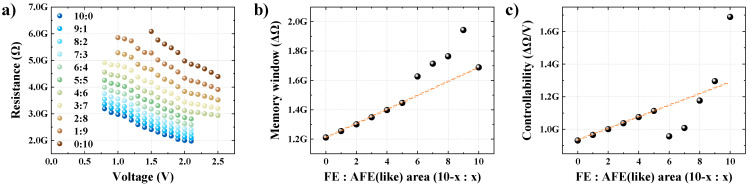
Evaluation of the resistance change memory characteristics; (**a**) resistance change characteristics considering stability; (**b**) size of memory window considering stability; (**c**) controllability considering stability.

Figure 5a shows the resistance change characteristics of ferroelectric and antiferroe-lectric (like) depending on the area ratio, considering the linearity of the resistance states. The case in which the resistance increased despite the increase in the amplitude of the applied pulse was excluded from the analysis. In addition, it was analyzed only when there was a change in the resistance state of 1% or more based on the memory window. The voltage regions used for analysis according to each ratio are as follows: resistance from 0.8 V to 2.1 V when x was 0 to 5, resistance from 0.8 V to 2.5 V when x was 6 to 7, resistance from 1.0 V to 2.5 V when x was 8 to 9, and resistance from 1.5 V to 2.5 V when x was 10, where x represents the ferroelectric ratio (ferroelectric:antiferroelectric (like) = 10-x:10). The memory window size and controllability, which are the two main parameters of the extracted multiresistance states, can be confirmed in Figure 5b,c, respectively. As shown in Figure 5b, the memory window size tends to increase as the area ratio of the antiferroelectric (like) increases. However, if the area ratio increases to 0:10, which corresponds to a single antiferroelectric (like), the memory window is reduced. This is due to the instability of the antiferroelectric (like) that occurs. It can be confirmed that the memory window size is advantageous at x = 7, 8, and 9, except for x = 10 when the ratio of the antiferroelectric (like) is high. The controllability characteristic of the multiresistance implementation is shown in Figure 5c. At an area ratio of 10:0 to 5:5, the size of the memory window increases; however, the voltage area of the applied pulse, in which the resistance changes is the same, and thus, the controllability parameter increases. This means that the controllability gradually deteriorates from 10:0 to 5:5. However, from 4:6 to 2:8, when the area ratio of the antiferroelectric (like) relatively increases, the controllability parameter decreases. This is because the voltage-controllable area that modulates the resistance state increases simultaneously with an increase in the memory window size. If the ratio of the antiferroelectric (like) increases further, the controllable voltage area decreases, and the controllability parameter increases, thus losing the advantage of controllability. Thus, it was determined that a ferroelectric:antiferroelectric (like) area ratio of 3:7 was the most beneficial for implementing multiresistance states. The memory window increased by 42%, and the controllability showed an advantage of 76.6 MΩ per 1 V compared to the single ferroelectric used for general FTJ. This result suggests that the limitation in implementing the multiresistance states of the existing FTJ can be overcome when the ferroelectric:antiferroelectric (like) has an area ratio of 3:7.

## 4. Conclusions

Ferroelectric and antiferroelectric (like) parallel structure based FTJ devices were proposed to overcome the multiresistance state limitations of the existing resistance change memory. The switching characteristics of each ferroelectric and antiferroelectric (like) device were anticipated using the schematic EBD, and this was confirmed through measurements. Through the characterization analysis of various parallel structures of ferroelectrics and antiferroelectrics, it was demonstrated that they have a larger memory window range than single ferroelectrics and can compensate for the stability problem of antiferroelectrics in parallel operation. In particular, it was presented that multiple resistance states can be stably controlled by adjusting the ferroelectric:antiferroelectric (like) area ratio. These results suggest that the limitation of implementing multiple resistance states in existing ferroelectricity may be overcome by driving ferroelectricity and antiferroelectricity (like) simultaneously. In conclusion, it is anticipated that the characteristics of the resistance change memory will be improved by integrating ferroelectric and antiferroelectric (like) materials in a parallel structure in a single device.

### Experimental

Characterization: Current mapping of the 10 nm-thick Hf_0.35_Zr_0.65_O_2_ ferroelectric thin film and 6 nm-thick Hf_0.35_Zr_0.65_O_2_ antiferroelectric (like) thin film were performed using AFM (XE7, Park Systems). An optional C-AFM module (Ultra-Low Current Amplifier (ULCA), Park Systems) was also used to amplify low-level currents. Image analysis software (XEI, Park Systems) was used to analyze the scanned images and process the data.

*Electrical Measurements*: Electrical measurements were performed using a parameter analyzer (4200A-SCS, Keithley) with a 4225-PMU. For both devices, pulses and DC signals were applied to the top electrodes, and the bottom electrodes were grounded. The low-level current was measured using a preamplifier connected to the SMU. All measurements were performed at room temperature.

## Data Availability

The data presented in this study are contained within the article.
